# Supplementary material for: Trust as a mediator in the relationship between childhood sexual abuse and IL-6 level in adulthood
Source: PLoS One. 2020 May 15;15(5):e0232932. doi: 10.1371/journal.pone.0232932 (PMC7228092; doi:10.1371/journal.pone.0232932)
Supplement: Attachment — Submitted filename: Response to reviewers.docx [file pone.0232932.s001.docx]

Dear Editor and Reviewer,

We thank you for your kind consideration and thoughtful feedbacks to our manuscript! Here are our responses to your comments, which are underlined.

To Editor:

1. Numbering of pages and lines – We added the pages and lines

2. Tables and Tables citations – Tables are included into the manuscript

3. Data availability – Yes, we can make the data available in a repository upon acceptance.

Journal Requirements:

1. Please ensure that your manuscript meets PLOS ONE's style requirements – Yes, we adjusted the format in accordance with PLOS ONE’s style requirements.

2. Please include a copy of Table 1-4 which you refer to in your text on page 4 and 7. – We included the tables.

To reviewer:

`

1- Please add all leading zeroes in numbers (0.01 vs .01). – Yes, we did.

2- Include tables showing the statistical outcomes of other measures (trauma, emotional abuse, etc). – Yes, we did.

3- Include reference for the following statement "CSA also seems to coexist with many other types of abuse (e.g. emotional, physical, domestic, neglect, etc.)."- – Yes, we did.

4- Include reference for the following statement "On one hand, this is understandable because CSA is considered to be the most traumatic of these childhood experiences, and can have stronger long-term negative impacts."- – Yes, we did.

5- Unless you have data relating to this statement: "At their current stage of personal development, CSA had only a trivial impact on these participants" do not include. – We deleted this statement.

6- The discussion states that "The above findings were not observed in participants who were not clinically depressed" Please elaborate more on this, explaining what the results were in those who were not clinically depressed. Include additional information relating to the role of IL6 and depression. – We added the elaboration.

7- Update figure one to read "Trust IN partner" not "Trust TO partner" as per the figure description – Yes, we revised the figure caption.

We thank you again for your time.
